# Early exercise after spinal cord injury (‘Switch-On’): study protocol for a randomised controlled trial

**DOI:** 10.1186/1745-6215-16-7

**Published:** 2015-01-07

**Authors:** Mary P Galea, Sarah A Dunlop, Ruth Marshall, Jillian Clark, Leonid Churilov

**Affiliations:** Department of Medicine (Royal Melbourne Hospital), The University of Melbourne, Parkville, VIC 3010 Australia; School of Animal Biology (M317), The University of Western Australia, Crawley, WA 6009 Australia; South Australian Spinal Cord Injury Service, Hampstead Rehabilitation Centre, 207-255 Hampstead Road, Northfield, SA 5085 Australia; Florey Institute of Neurosciences and Mental Health, Melbourne Brain Centre (Austin Campus), Heidelberg, VIC 3084 Australia

**Keywords:** Spinal cord injury, Randomised controlled trial, Early exercise, FES cycling, Passive cycling, Muscle atrophy, Biomarkers, Osteoporosis, Neurological function, Immune function, Quality of life

## Abstract

**Background:**

Spinal cord injury (SCI) leads to a profound muscular atrophy, bone loss and bone fragility. While there is evidence that exercising paralysed muscles may lead to reversal of muscle atrophy in the chronic period after SCI, there is little evidence that exercise can prevent muscle changes early after injury. Moreover, whether exercise can prevent bone loss and microarchitectural decay is not clear.

**Methods/Design:**

A multi-centre, parallel group, assessor-blinded randomised controlled trial will be conducted. Fifty participants with acute spinal cord injury will be recruited from four SCI units in Australia and New Zealand. Participants will be stratified by site and AIS status and randomised to an experimental or control group. Experimental participants will receive a 12-week programme of functional electrical stimulation (FES)-assisted cycling. Control participants will receive a 12-week programme of passive cycling. The primary outcome is muscle cross-sectional area of the thigh and calf measured using magnetic resonance images (MRI) of the leg. Secondary outcomes include serum biomarkers of SCI osteoporosis (sclerostin, P1NP and β-CTX), markers of immune function (IL-6, IL-10, FGF2, INF-γ, TNF-α), neurological function, body composition, depression and quality of life. Leg MRIs will be measured by a single blinded assessor based in Melbourne. Serum samples will be analysed in a central laboratory. All other characteristics will be measured at baseline and 12 weeks by blinded and trained assessors at each site. The first participant was randomised on 27 November 2012.

**Discussion:**

The results of this trial will determine the relative effectiveness of a 12-week programme of FES-assisted cycling versus passive cycling in preventing muscle atrophy and maintaining skeletal integrity after spinal cord injury.

**Trial registration:**

ACTRN12611001079932 (18 October 2011)

## Background

Spinal cord injury (SCI) leads to a profound muscular atrophy, bone loss and bone fragility [1–3]. Fractures lead to a marked deterioration in bone quality, and compromise rehabilitation and quality of life [4].

Furthermore, recent advances have identified mechanisms that link muscle and bone metabolism, thereby identifying such tissues as endocrine organs [5, 6]. The discovery that muscle-secreted factors contribute to osteocyte viability [7] suggests a link between disuse and endocrine functions of bone and between muscle endocrine action and susceptibility for chronic disease [8, 9]. Such developments have highlighted inter-relationships between the aetiologies of sarcopenia and osteopenia [10], cardinal features of secondary disuse disorder such as occurs after SCI [11, 12]. Recognition that SCI involves an ‘endocrine syndrome’ [13, 14] has advanced our understanding of regulatory mechanisms that contribute to the clinical features of this catastrophic injury. Given that muscle-secreted factors (myokines) have endocrine and other actions, the role of skeletal muscle contraction induced by exercise following SCI warrants closer attention.

Thigh girth reduces by 50% within 3 weeks of SCI [15]; thickness of muscle bulk, measured using ultrasound, reduces by 40% in the first month [16]. A magnetic resonance imaging (MRI) study of changes in muscle cross-sectional area (CSA) over the first few months after SCI showed that the mean CSA of the quadriceps, hamstring and adductor muscle groups declined by 16%, 14% and 16% respectively [17].

Muscle is a dynamic tissue capable of adapting its morphology depending on the functional demands placed on it. There is evidence that exercising paralysed muscles through functional electrical stimulation (FES)-assisted cycling leads to reversal of atrophy [18]. Furthermore, in rats with spinal transection, passive exercise in the form of pedaling on a motor-driven cycle results in activation of muscle satellite cells and reversal of hindlimb muscle atrophy. This effect is more marked in muscles with slow oxidative fibres such as soleus [19].

It is still unknown whether unloading of the bones in the paralysed extremities is the only factor causing rapid bone loss below the level of injury, or if other factors such as neuronal, humoral and vascular adaptations after a SCI are also involved [20, 21]. Muscle forces create the peak forces acting on bone, a relationship summarised by the mechanostat theory [22], which predicts that bone mass, strength and size will respond to increasing muscle maximal forces during bone growth, or the loading of mature bone. Unloading through disuse or immobilisation will have a negative effect on bone mass and strength, both through muscle atrophy and local regulators of cell phenotype, within the bone tissue microenvironment.

Sclerostin, encoded by the *sost* gene, is a Wingless-Int (Wnt) signalling pathway antagonist secreted by osteocytes and is a potent inhibitor of bone formation [23]. Compared to other bone-related proteins, sclerostin is considered a candidate biomarker for SCI-induced osteoporosis [24]. Sclerostin is increased by unloading and suppressed by loading, a feature observed at least in the first few years after SCI [25, 26]. In addition to the mechanical loading of bone through muscle contraction, muscle myokines released from contracting muscles may protect osteocytes from undergoing cell death [27].

One study has used FES early after injury to *prevent* muscle changes rather than *reverse* them when losses are well advanced. Baldi *et al*. [12] compared FES-assisted cycling with FES-induced isometric muscle contractions commencing within the first 12 to 15 weeks of injury. Control subjects who had had no exercise lost 21 to 27% of muscle mass. FES-cycling, but not FES-induced isometric exercise, prevented loss of lower limb lean body mass and gluteal lean body mass at both 3 and 6 months.

Whether exercise can *prevent* bone loss and microarchitectural decay or reverse it when established is not clear. A regimen of FES-assisted isotonic contractions instituted early after SCI did not lead to an osteogenic effect in the lower limbs, measured using dual energy X-ray absorptiometry [28]. A 2010 systematic review [29] has concluded that FES-cycling does not improve or maintain bone at the tibial diaphysis in the acute phase, but may increase/maintain lower extremity bone mineral density (BMD) in the chronic phase after SCI [30], the implication being that site-specific bone adaptation is both time- and dose-dependent and thus, mechanisms of bone adaptation may differ in acute and in established SCI.

In this trial, we will provide an exercise programme that involves FES-cycling against increasing loads as early as practicable after SCI, commencing when the individual is still confined to bed. We hypothesise that muscle activation will prevent muscle atrophy. In addition, we hypothesise that mechanical strains associated with loading and changing leg position during cycling will inhibit sclerostin levels and thereby preserve skeletal structure by reducing inhibition of bone formation and reduce increased bone resorption at the cellular level seen early after injury.

## Methods

The trial was registered on the Australian and New Zealand Clinical Trials Registry (ACTRN12611001079932) on 18 October 2011. The first participant was randomised on 27 November 2012, and the final participant on 31 March 2014.

### Funding

The study is being funded by the Transport Accident Commission (Victorian Neurotrauma Initiative), and the National Health and Medical Research Council of Australia. At the conclusion of the trial, all equipment purchased for the trial will be gifted to the participating hospitals.

### Design

A multi-centre, assessor-blinded, randomised controlled trial will be conducted. Experimental group participants will undertake an intensive 12-week programme of FES-assisted cycling, while control group participants will undertake a passive cycling programme over the same period. The trial will be conducted in four spinal units in Australia and New Zealand. Ethical approval has been obtained from the Human Research Ethics Committees at Austin Health in Melbourne (H2012/04476), Royal Perth Hospital in Perth (EC 2012/080), Princess Alexandra Hospital in Brisbane (HREC/12/QPAH/085) and the Multi-region Ethics Committee in New Zealand (MEC/12/02/020) and at the University of Melbourne (Trial Sponsor, HREC 1237599). Participants will be provided with information sheets and written informed consent will be obtained prior to recruitment and baseline assessment. See Table 1 for the schedule of enrolment, interventions, and assessments.

**Table 1:** **Standard Protocol Items: Recommendations for Interventional Trials (SPIRIT) checklist: schedule of enrolment, interventions, and assessments**

	Enrolment	Allocation	Intervention	Post-intervention
TIMEPOINT	***-t***_***1***_	0	12 weeks	12 weeks
**ENROLMENT:**				
**Eligibility screen**	X			
**Informed consent**	X			
**Medical clearance**	X			
**Allocation**		X		
**INTERVENTIONS:**				
***FES-assisted cycling***				
***Passive cycling***				
ASSESSMENTS*:				
***Muscle cross-sectional area of thigh and calf (1° outcome measure)***	X			X
***Serum sclerostin***	X			X
***Other serum biomarkers***	X			X
***Muscle mass***	X			X
***ISNCSCI Motor and Sensory Scores***	X			X
***Depression (HADS)***	X			X
***Quality of Life***	X			X
***(HUI3, AQoL, WHOQoL-Bref)***				

*1-week window allowed for assessments.

FES = functional electrical stimulation; ISNCSCI = International Standard for Neurological Classification of Spinal Cord Injury; HADS = Hospital Anxiety and Depression Scale; HUI3 = Health Utilities Index Mark 3; AQoL = Assessment of Quality of Life; WHOQoL-Bref = WHO Quality of Life-Bref.

### Participants

Fifty participants with new acute SCI admitted to one of the four trial sites will be recruited.

### Inclusion criteria

Participants will be included if they:

1. Have sustained a complete or incomplete SCI above T12 no more than 3 weeks previously
2. Have undergone internal fixation of their spinal fracture or whose fracture is considered sufficiently stable
3. Are medically stable
4. Are able to provide informed consent
5. Have medical and surgical clearance to participate in the study

### Exclusion criteria

1. Under 18 years of age
2. American Spinal Injury Association Impairment Scale (AIS) D (50% of the muscles below the injury level are scored as 3 or above) at baseline
3. In halo or other traction
4. In the ICU
5. Pressure ulcers
6. Peripheral nerve lesion
7. Long bone or pelvic fracture
8. Lower limb amputation
9. Metabolic bone disease, including lytic or renal bone disease, or senile osteoporosis
10. Exposure to drugs that affect bone metabolism (amino-bisphosphonate, high-dose glucocorticoids, cyclosporine, anti-epileptic drugs (AEDs))
11. Known contraindications to FES (cardiac pacemaker, epilepsy, pregnancy)
12. Any other contraindications to participating in exercise programmes, or outcome assessments, as advised by the treating physician
13. English language competency insufficient to understand research procedures and provide informed consent.

### Randomisation

Participants will be randomly assigned to either the experimental or control group with a 1:1 allocation as per a computer-generated randomisation schedule stratified by participating site and injury status (AIS) A/B or AIS C). The randomisation schedule will be under the control of a central randomisation unit, independent of the trial, located at Neuroscience Trials Australia in Melbourne. A participant will be considered to have entered the trial once his/her randomisation is revealed to the site coordinator.

### Intervention

All participants will attend the assigned exercise programme for 48 sessions over 12 weeks, with an intended frequency of 4 times weekly.

#### Experimental group

Participants in the experimental group will receive FES-assisted cycling using a Hasomed RehaStim FES Unit attached to a MOTOmed cycle (Hasomed GmbH, Magdeburg, Germany) while they are confined to bed, and an RT300 cycle (Restorative Therapies, Baltimore, MD, USA) when they are able to sit in a chair. Surface electrodes will be applied over the belly of the quadriceps, gluteal, and hamstrings muscles according to a standardised protocol [31]. The pedal cadence will be set to 15 to 50 rev.min^−1^ Stimulation intensity will be gradually increased to a maximum of 140 mA with a pulse width of 0.3 ms at a frequency of 35 Hz [31, 32]. Participants will exercise at the maximal power output possible at their level of recovery. Therapists will evaluate the degree of muscle contraction, power output and fatigue, and adjust settings appropriately to maximise time, intensity, and power of training with a principle of overloading the muscle. When fatigue occurs, cycling can be stopped and then re-commenced after a 5-minute break if muscle contraction can be re-elicited. For participants with some degree of volitional muscle activity, the settings will be adjusted to ensure that resistance is maintained.

Participants will undertake 30 minutes of cycling on 4 days/week for 12 weeks, initially in intermittent periods of exercise with rest breaks, but progressing to a maximum of 60 minutes as deemed appropriate by the supervising therapist/assistant. This can be undertaken as a single session or split into two sessions, depending on the participant’s performance and the availability of staff. All aspects of each training session will be recorded.

#### Comparator group

Participants allocated to this group will undertake up to 60 minutes passive cycling on 4 days/week for 12 weeks at identical pedal cadence to the Experimental Group. Similar to the Experimental Group, cycling while the participant is confined to bed will be with the MOTOmed Letto device (Hasomed GmbH, Magdeburg, Germany) and, once mobilised into a wheelchair, may use the RT300 cycle (Restorative Therapies, Baltimore, MD, USA) but without FES-evoked contractions.

#### Follow-up period

All participants will complete a post-intervention visit at the end of the 12-week intervention period. Assessments completed at baseline will be repeated. A follow-up phone call will be made 1 month after this visit to check on progress or adverse events in the intervening period.

#### Quality assurance

To ensure that treatments are of a high standard and delivered in accordance with the trial protocol, therapists responsible for administration of the exercise programmes will attend workshops where they will be trained in the delivery of the treatment programme and in assessment procedures. Therapists will also be provided with a written protocol and standardised case report forms for documentation of assessments and interventions.

### Assessments

Assessments will occur at baseline, and at 12 weeks after the commencement of the intervention. All assessments will be undertaken by therapists blinded to group allocation. Any inadvertent unblinding of assessors will be reported.

### Primary outcome

#### The primary outcome measure is muscle cross-sectional area of the thigh and calf at 12 weeks

Magnetic resonance images of the leg and thigh will be collected with a 1.5-Tesla magnet. Fifteen transaxial images, 1 cm thick and spaced 0.5 cm apart will be taken from the knee joint to the ankle joint (leg) and from the hip joint to the knee joint (thigh) using a whole-body coil. Markings on the mid-leg and mid-thigh aligned with cross-hairs on the imager will allow for similar positioning during repeat scans. The participants’ feet will be strapped to a brace to maintain the ankle joint at approximately 90° and the knee and hip joints extended. Participants will wear anti-embolism stockings for 30 minutes prior to imaging to minimise fluid shifts in the leg.

### Secondary outcome measures

To control for sources of pre-analytical variability, participants will be required to fast for 8 hours before blood is drawn between 0800 and 0900 hours (35 ml per sample). Serum samples will be collected at baseline, 1, 2, 3, 4, 8 and 12 weeks (end of intervention). All samples will be separated and stored at ─20°C until assayed in a central laboratory, and will be destroyed using standard laboratory techniques after the completion of the study.

### Serum sclerostin

Serum sclerostin will be measured using ELISA incorporating a biotinylated antibody/horseradish peroxidase-streptavidin for detection of the analyte. The detection limit of the sclerostin ELISA is 0.18 *mg.mL*^*−1*^ and the intra-assay coefficient of variability (CV) is between 6 to 10% [33].

### Other serum markers

The bone biomarkers N-propeptide of type I procollagen (P1NP) and β-isomerised C-terminal telopeptide of collagen type I (β-CTX) will be measured using the electrochemiluminescence sandwich immunoassays (both Elecsys 1010 Analytics, Roche Diagnostics, Mannheim, Germany).

The total P1NP assay detects the monomer and trimeric forms of the molecule represented in the serum. The reference standards given by the manufacturer for this assay (imprecision 1.8 to 2.9%) independently were validated (1.4 to 2.3%) ([34]. The inter-assay CVs are 1.4%, 1.7% and 2.0% at 74 μ*g.L*^*−1*^, 508 μ*g.L*^*−1*^ and 978 μ*g.L*^*−1*^, and intra-assay CVs are 2.0% and 2.3% at 47 μ*g.L*^*−1*^ and 643 μ*g.L*^*−1*^[34].

The monoclonal antibodies used in the Elecysis B-Cross Laps assay recognise all fragments of the β-δAA octapeptide of type 1 collagen. Intra-assay precision (CVs) given by the manufacturer is 4.6%, 1.8% and 1.0% at 0.08 *ng.mL*^*−1*^, 0.39 *ng.mL*^*−1*^ and 3.58 *ng.mL*^*−1*^. Total precision (CV) is 4.7%. 4.3% and 1.6% at 0.08 *ng.mL*^*−1*^, 0.39 *ng.mL*^*−1*^ and 3.58 *ng.mL*^*−1*^ respectively.

Biomarkers of immune function (systemic low-level inflammation), which also are myokines, will be assessed using a MILLIPLEX MAP Human Cytokine/Chemokine Magnetic Bead Panel Immunology Multiplex Assay read with a Luminex xMAP® platform (Merck Millipore, Darmstadt, Germany) in a magnetic bead format for the determination (*pg.ml*^*−1*^) of: IL-1β, IL-6, IL-10, IL-15, INF-γ, fibroblast growth factor 2 (FGF2), CX3CL1 (fractalkine), CCL2 (monocyte chemotactic protein-1: MCP-1), TNF-α, vascular endothelial derived growth factor (VEGF) and brain-derived neurotrophic factor (BDNF). Precision data given by the manufacturer estimate accuracy as 87 to 107%. Intra-assay and inter-assay CVs are given as 2 to 13% and 5 to 19%, respectively.

### Muscle mass

Muscle mass will be determined using standard anthropometric methods (measurement of lower limb girths and skinfold thickness) [35] according to the International Society for the Advancement of Kinanthropometry (ISAK) standards. The intraobserver reliability of anthropomentry is high (R > 0.9) [36].

### ISNCSCI motor and sensory scores

Motor and sensory recovery will be measured using the International Standard for Neurological Classification of Spinal Cord Injury (ISNCSCI) [37]. The motor score is derived from testing strength of five key upper limb and five key lower limb muscles bilaterally with the subject positioned in the supine position (for example, elbow flexors, wrist extensors, hip flexors, quadriceps, dorsiflexors) on a scale of 0 = no contraction to 5 = normal resistance through full range of motion. Scores are summed to give a total possible score of 50 for the upper extremities and 50 for the lower extremities. The sensory test involves testing pin-prick and light-touch sensation at key points representing each dermatome. Pin-prick and light-touch sensation of each dermatome are separately scored on a 3-point scale (0, 1 and 2). Scores will be summed to give a total possible score of 224, where a higher score indicates better sensation than a lower score. The total ISNCSCI motor, light-touch and pin-prick scores are highly reliable when assessed by experienced examiners [38].

This assessment will be used during the baseline visit to confirm the participant’s motor, sensory and neurological levels as well as the AIS classification, which indicates the degree of completeness of the spinal cord lesion prior to randomisation and then again at the post-intervention assessment.

### Depression

Depression will be measured using the Hospital Anxiety and Depression Scale (HADS) [39], which has been found to be valid [40] and reliable [41] for people with SCI.

### Assessment of Quality of Life (AQoL-8)

The AQoL-8 is a multi-attribute utility instrument. It will be used to evaluate whether the study intervention improves participants’ quality of life as well as for an economic evaluation of the intervention’s cost-effectiveness. The AQoL-8 is a self-administered 8-item questionnaire [42] with excellent test-retest reliability [43].

### Health Utilities Index Mark 3 (HUI-3)

HUI-3 is a measure of health-related quality of life widely used in population health surveys, clinical studies and cost-utility analyses. HUI-3 includes eight attributes (vision, hearing, speech, ambulation, dexterity, emotion, cognition and pain), with five or six levels for each attribute. HUI-3 is reliable [44] and is able to discriminate various aspects of burden associated with chronic conditions as well as describing the differences in overall health-related quality of life levels [45].

### WHOQoL-Bref

The WHOQoL-Bref questionnaire was designed by an international collaboration on quality of life by the World Health Organisation. It contains 26 questions about many different aspects of quality of life, with some additional questions about the person and his/her health. It has good test-retest reliability [46] and has been found to be a valid measure in people with SCI [47].

### Statistical analyses

#### Sample size

The power analysis is for a one-way fixed effects analysis of covariance (ANCOVA) with two levels that correspond to FES cycling and passive cycling arms of the study. This analysis includes baseline value of muscle CSA in the thigh and calf as a covariate, and uses the value of muscle CSA in the thigh and calf at week 12 as the primary outcome. Based on available pilot data [17], the participants in the passive cycling arm are likely to lose around 33% of the baseline muscle CSA (mean = 75, SD = 20) over a period of 12 weeks after SCI (measured using MRI). Assuming this loss could be contained to 10% by the proposed FES cycling intervention, thus resulting in mean changes of 25 (SD = 20) and 7.5 (SD = 20) for passive cycling and FES cycling respectively, the total sample size of 42 patients equally distributed between FES cycling and passive cycling arms will yield power of 0.8 to reject the null hypothesis at 0.05 level of significance. The ANCOVA is conservative (that is assuming no correlation between the baseline and follow-up measures) and non-directional (that is two-tailed) which means that an effect in either direction will be interpreted. Allowing for a15% drop-out rate, the final sample size for this study is calculated to be 50 patients equally distributed between FES cycling and passive cycling arms.

### Statistical analysis

The primary and secondary endpoint analyses will be conducted on an intention-to-treat basis. Baseline comparisons between the 2 arms of the study will be made using means or medians and proportions with 95% confidence intervals. All endpoints and analyses have been prospectively categorised as either primary or secondary. Differences in both primary and secondary endpoints between the 2 arms of the study will be tested independently at the 0.05 level of significance, using a one-way ANCOVA model that will include the baseline value of a given outcome in question as a covariate. No formal adjustments will be undertaken to constrain the overall type I error associated with the secondary analyses. Their purpose is to supplement evidence from the confirmatory primary analysis to help more fully characterise the treatment effect. Results from the secondary analyses will be interpreted in this context.

Additional exploratory analyses of the effects of stratification factors on the differences in both primary and secondary endpoints between the two arms of the study will be undertaken by either explicitly including these factors into the model (injury status) or by treating them as random effects in a random-effect regression model (study sites).

### Data integrity and management

Data will be stored electronically on a database with secured and restricted access. Data transfer will be encrypted and any information capable of identifying individuals removed.

### Withdrawals

A participant will be considered to have withdrawn from the trial when consent is revoked or if the participant cannot be contacted or located. If this occurs, no further assessments will be performed. Participants will not be withdrawn from the trial for protocol violations.

### Monitoring

The trial will be overseen and monitored by a Programme Manager. The Programme Manager will visit each site to examine trial procedures, ensure data quality and monitor compliance with the trial protocol. All adverse events and serious adverse events will be recorded according to standard procedures. An interim safety analysis is planned when 25 participants have completed their post-intervention assessment. It will be done by an independent Data Safety Monitoring Board (DSMB) comprising a statistician and two rehabilitation doctors. If there are concerns about the safety of participants, this board will make a recommendation to the trial steering committee about continuing, stopping, or modifying the trial.

## Discussion

This trial will provide information about the relative effectiveness of FES-assisted cycling versus passive cycling in preventing muscle atrophy. This is important because muscle atrophy is associated with blood vessel atrophy [48] and predisposes to the development of pressure ulcers. The mechanisms by which active and passive exercise programmes improve function after SCI also need to be better understood because they allow for development of new and more effective therapeutic strategies. In this study, the control intervention is not standard care but an active intervention, involving passive cycling. This ensures that the intensity of both exercise programmes is similar, so that the effects of active exercise of the paralysed lower limbs can be identified over and above passive movement of the limbs.

This trial will adhere to key methodological principles important for minimising bias (International Conference on Harmonisation Good Clinical Practice annotated by the Therapeutic Goods Administration of Australia) [49] and will be reported according to the CONSORT guidelines [50]. For example, allocation will be concealed and randomised, assessors will be blinded and analyses will be performed on an intention-to-treat basis. Therapists and participants will not be blinded due to the nature of the intervention. One primary outcome and a number of secondary outcomes will be used. The primary outcome is a measure of muscle CSA. The secondary outcomes include measures of a biomarker for disuse osteoporosis, body composition, neurological function, and quality of life. Importantly, all adverse events will be rigorously documented, so that the safety of the interventions can be evaluated. It is anticipated that this trial will take 24 months to complete.

## Trial status

Recruitment commenced in October 2012. Recruitment continued until 31 May 2014.

## Abbreviations

AED: Anti-epileptic drugs
AIS: American Spinal Injuries Association Impairment Scale
ANCOVA: Analysis of covariance
AQoL-8: Assessment of Quality of Life 8
β-CTX: β-isomerised C-terminal telopeptide of collagen type I
BDNF: Brain derived neurotrophic factor
BMD: Bone mineral density
CCL2: Monocyte chemotactic protein-1(MCP-1)
CONSORT: Consolidated Standards of Reporting Trials
CSA: Cross-sectional area
CV: Coefficient of variation
CX3CL1: Fractalkine
DSMB: Data Safety Monitoring Board
ELISA: Enzyme-linked immunosorbent assay
FES: Functional electrical stimulation
FGF2: Fibroblast growth factor 2
HADS: Hospital Depression Anxiety Scale
HUI-3: Health Utilities Index Mark 3
IL: Interleukini
INF: Interferon
ISAK: International Society for the Advancement of Kinanthropometry
ISNCSCI: International Standard for Neurological Classification of Spinal Cord Injury
MRI: Magnetic Resonance imaging
P1NP: N-propeptide of type I procollagen
SD: Standard deviation
SCI: Spinal cord injury
SPIRIT: Standard Protocol Items: Recommendations for Interventional Trials
TNFα: Tumour necrosis factor alpha
VEGF: Vascular endothelial derived growth factor
WHOQoL-Bref: World Health Organisation Quality of Life Short Form
Wnt: Wingless-related integration site gene.
